# Dietary Determinants of Anemia in Children Aged 6–36 Months: A Cross-Sectional Study in Indonesia

**DOI:** 10.3390/nu13072397

**Published:** 2021-07-13

**Authors:** Diana Sunardi, Saptawati Bardosono, Ray W. Basrowi, Erika Wasito, Yvan Vandenplas

**Affiliations:** 1 Department of Nutrition, Faculty of Medicine Universitas Indonesia, Cipto Mangunkusumo General Hospital, Jakarta 10430, Indonesia; diana_sunardi@yahoo.com (D.S.); tati.bardo@yahoo.com (S.B.); 2 Medical Nutrition for Danone Specialized Nutrition, Yogyakarta 55165, Indonesia; ray.basrowi@gmail.com (R.W.B.); erika.wasito@danone.com (E.W.); 3 Vrije Universiteit Brussel (VUB), UZ Brussels, KidZ Health Castle, 1090 Brussels, Belgium

**Keywords:** anemia, cow’s milk, cow’s milk formula, zinc, toddler, Indonesia

## Abstract

Anemia has been acknowledged as worldwide problem, including in Indonesia. This cross-sectional study aims to explore dietary determinants as risk factors for anemia in children aged 6–36 months living in a poor urban area of Jakarta. The study was done in Kampung Melayu sub-district in Jakarta, Indonesia. Data was collected within two weeks in September–October 2020. A structured questionnaire for a 24-h recall and a semi-quantitative Food Frequency Questionnaire (FFQ) were used to collect the dietary intake data, and venous blood was withdrawn to determine the hemoglobin levels. Bivariate chi-square and multiple logistic regression tests were executed to explore the dietary determinant factors for anemia. We recruited 180 subjects. The average hemoglobin concentration was 11.4 ± 1.7 mg/dL; the anemia prevalence was 29.4%. The following variables were significantly associated with higher risk of anemia: no cow’s milk formula consumption, inadequate intake of fats, protein, calcium, vitamin D, iron, zinc, vitamin A, vitamin C, vitamin B6, and vitamin B12. Only cow’s milk formula consumption and zinc intake were revealed as the determinant factors of anemia. In conclusion, the prevalence of anemia was 29.4% among children aged 6–36 months old. Anemia was significantly associated with two dietary determinants as risk factors that are cow’s milk formula consumption and zinc intake.

## 1. Introduction

Anemia has been acknowledged as a worldwide health problem that young children are specifically vulnerable. The data from the World Health Organization (WHO) shows that anemia prevalence in children aged 6–59 months in Indonesia are 43.9% in 2000 and 38.4% in 2019 [1]. A similar anemia prevalence (38.5%) is also reported from Indonesian national data in 2018 [2]. Another study in Indonesian rural area in 2009–2010 showed that the prevalence of anemia and iron deficiency anemia (IDA) in children aged 6–59 months were 56.9 and 29.4%, respectively [3]. The prevalence was higher than the WHO data in 2000 or the latest national data in 2018 that might indicate higher risk of anemia in the rural area. Childhood anemia contributes to poor motor and cognitive development resulting in poor school performance, and results in increased morbidity and mortality [4].

There are two types of anemia: nutritional and non-nutritional related. In nutritional anemia, there is insufficient intake of nutrients to meet the need for hemoglobin and erythrocyte synthesis. Special attention needs to be given to the consumption of iron-rich or iron fortified foods because iron deficiency is the common cause of anemia among under-five year old children [5]. It is estimated to contribute to 42% of anemia cases in under 5-year-old children worldwide [6]. Other nutrients that contribute to anemia are deficiencies of vitamin A, B2 (riboflavin), B6 (pyridoxine), B12 (cobalamin), C, D, E, folate, and copper [6]. Most anemia studies in under-five-year-old children highlighted the relation with maternal factors, socio-economic factors, and failure to thrive related factors. A systematic review found that poor dietary diversity is one of the predictors for anemia in under-five-year-old children, along with failure to thrive, food insecurity and not being dewormed [7]. A study in Indonesia in 2017 found that the small quantity of lipid-based nutrient supplement was effective in improving the hemoglobin level and reduced the incidence of anemia in infants aged 6–12 months after the three-month intervention period [8]. The study showed that this supplement could fill the gap of iron intake as it contained 6 mg Fe and 30 mg vitamin C [8]. This shows the importance of dietary intake as a determinant factor in childhood anemia. Therefore, this new study aims to explore dietary determinant as risk factors of anemia among children aged 6–36 months living in a poor urban area of Jakarta.

## 2. Materials and Methods

Study design. This study is an observational analytical cross-sectional study.

Location and time. The poor urban Kampung Melayu sub-district in Jakarta, Indonesia, was purposively selected because it was the only area permitted by the local authority while other areas were closed due to COVID-19. Data collection was done within two weeks in September–October 2020, while strictly applying the COVID-19 health safety procedure.

Population and sample. Children aged 6–36 months were recruited from the selected Posyandu (i.e., community health post) after obtaining the signed informed consent from their parents. Those children who were seriously ill and/or needed special medication were excluded. It was calculated that at least 80 subjects were needed as a minimal sample size, considering an anemia prevalence of 29.4%, with a 95% degree of significance (Z_alpha_ = 1.96) and 90% degree of reliability.

Data collection. Socio-demographic characteristics of the subjects, i.e., age, sex, general health status, parents’ education, and family income were collected using a structured questionnaire. Macronutrient intake was determined using a dietary intake assessment of a one-day 24-h recall, while for the micronutrient intake data was collected using semi-quantitative food frequency questionnaire (FFQ) over a period of the past two weeks [9]. Inadequate intake was defined as an intake that was below the Indonesian recommended daily allowance (RDA). Anemia was diagnosed using the cyanmethemoglobin method for venous blood to measure hemoglobin levels. The cut-off of hemoglobin level less than 11.0 g/dL is used, and we assumed that nutritional factors were likely to be the most important [10].

Data management and analysis. All data were recorded using a clinical record form before being entered into the spreadsheet using SPSS version 20.0. After data cleaning, data were analyzed using descriptive and inferential statistical tests to explore possible determinants of anemia using chi-square, and logistic regression analyses were performed in those with *p*-value < 0.020 according to the chi-square test [11]. A statistically significant level was determined using *p*-value less than 0.05.

Ethics: Data collection was done after receiving ethical approval released dated 27 April 2020 by the Ethical Committee Faculty of Medicine Universitas Indonesia (i.e., No. KET-438/UN2.F1/ETIK/PPM.00.02/2020) and obtaining informed consent from the parent.

## 3. Results

The subjects’ recruitment and data collection were permitted by the local authority for two weeks only because of safety reasons. During the COVID-19 pandemic, under-five year old children were not allowed to go outside their house. Even the monthly Posyandu for child health and nutrition monitoring was closed. Thus, we collaborated with the Posyandus’ volunteer health workers to screen the eligible subjects to participate in this study.

From the total of 185 participants (Table 1), we could not obtain a balanced inclusion according to age category, but sex distribution was similar. Regarding socio-demographic parental characteristics, the majority of fathers and mothers were mostly graduated from senior high school (64.9% and 58.9%, respectively) and had non-permanent jobs (61.6% and 96.8%, respectively), with a household income that was less than the recommended provincial minimal income (77.3%). These conditions matched the characteristics of a slum urban area in which the houses are very small and mostly rented with dense crowded neighborhoods.

Table 2 shows that the majority of subjects were reported to have exclusive breastfeeding experience for six months (78.4%), 63.2% subjects consumed cow’s milk growing-up formula, and only 21.1% subjects took vitamin-mineral supplements.

Data in Table 3 reveals that more than 50% of the subjects had insufficient dietary intake of energy, carbohydrate, fats, calcium, vitamin D, and folate according to the Indonesian RDA. Insufficient dietary intake of iron and vitamin C were found in 48.1% and 30.3% of the subjects, respectively.

As shown in Table 4, the mean hemoglobin level was 11.4 ± 1.7 mg/dL, and the lowest hemoglobin value was found among subjects aged 6–11 months (10.9 ± 1.6 mg/dL). The prevalence of anemia (i.e., hemoglobin less than 11.0 mg/dL) was 29.4%, and the highest prevalence was found among those aged 6–11 months (42.3%). Significant difference of hemoglobin level was found related to the fathers’ education and household income. However, there is no significant difference in anemia prevalence based on socio-demographic characteristics.

Using bivariate analysis (Table 5) to explore the dietary determinants of anemia among children aged 6–36 months, this study found significant associations between anemic status and inadequate dietary intake of fats (OR = 2.675), protein (OR = 3.3526), calcium (OR = 4.663), iron (OR = 3.681), zinc (OR = 3.960), vitamin A (OR = 4.525), vitamin C (OR = 2.797), vitamin B6 (OR = 2.860), vitamin B12 (OR = 3.290), and not consuming cow’s milk formula (OR = 9.849).

Based on further analysis using the logistic regression (Table 5) after controlling for fathers’ education and household income, this study found still two dietary determinant factors that significantly contributed to anemic status among children aged 6–36 months: inadequate dietary intake of zinc (OR = 4.262) and not consuming cow’s milk formula (OR = 8.651).

## 4. Discussion

The socio-demographic characteristics of the area in this study matched the characteristics of a slum urban area. The prevalence of inadequate intake was found almost for all macro and micronutrient intake, namely carbohydrate, fats, calcium, vitamin D, folate, iron, and vitamin C. The prevalence of anemia among children aged 6–36 months was 29.4%, in which zinc intake and absence of cow’s milk formula consumption were the key nutrient determinant factors that significantly associated with anemia.

The World Health Organization (WHO) has classified an anemia prevalence, estimated from serum levels of hemoglobin, between 20.0 to 39.9% as a significant moderate public health problem and a prevalence above 40% as of severe public health significance [10]. Thus, the anemia prevalence among children aged 6–36 months found in our study (29.4%) falls into the category of moderate public health significant. The prevalence of anemia in this study was lower than the reported prevalence in children aged 6–59 months in a rural area of Indonesia in 2014 (56.9%) [4] or the Indonesian national data in 2018 (38.5%) [2]. More concern should also be raised specifically for the age group 6–11 months because the prevalence of anemia in this age group reached 42.3%, what is classified by the WHO as of severe public health significance. The high anemia prevalence in this specific age group should also be carefully interpreted because the number of children in this age group was unfortunately much smaller than the other age groups. Furthermore, the study only provides a general description of anemia prevalence based on hemoglobin analysis with the assumption that nutritional factors were likely to be the most important factors. A shortcoming of our study design is the absence of other indicators of anemia, i.e., mean corpuscular volume (MCV), total count of erythrocytes, or reticulocyte count, thus limit the interpretation to a specific cause of anemia, i.e., iron deficiency anemia.

The high prevalence of anemia (41.1%) in a study done in Ethiopia was significantly associated with a number of variables, such as age group, living in an urban area, no formal education of mothers, primary education of mothers, low family monthly income, early introduction of complementary foods, and underweight [12]. Similarly, a cross-sectional study in the Lao People’s Democratic Republic in 2017 reported a prevalence of anemia among children aged <5 years of 43.0%, with three determinant factors identified: sex, underweight, and residence status [13]. Another cross-sectional study in Peru found that 53% children aged 6–35 months suffered from anemia that was associated with intestinal infection and poor access to safe drinking water [14]. In our study, the prevalence of anemia was not significantly associated with socio-demographic characteristics, but significantly associated with insufficient dietary intake of almost all relevant nutrients [15], i.e., more than 50% of inadequate dietary intake of fats, calcium, vitamin D, and folate intake, and more than 30% of inadequate dietary intake of iron, zinc, and vitamin C intake, also more than 15% inadequate intake of protein vitamin A, vitamin B6, and vitamin B12.

Due to the time restriction to conduct the study, it was not possible to conduct multiple 24-h recalls nor validate the semi-quantitative FFQ in this study. However, based on the FAO dietary assessment guideline, a one-day 24-h recall is still relevant to determine the mean intakes for a specific group or a population, i.e., children aged 6–36 months in this study. Given the time restriction, the semi-quantitative FFQ in this study was adapted from the existing one by adjusting the food list to target specific nutrients, which were known related to anemia. The existing semi-quantitative FFQ with Indonesian food list is commonly used in Indonesia, specifically for under-five-year-old children. The FFQ has the strength to capture a range of foods, specific nutrient(s), or a specific food group, including rarely consumed food items [16].

Parents had a sufficient level of education (i.e., graduated from senior high school), but more than 60% of the fathers and almost all the mothers had non-permanent jobs. Aligned with the parents’ situation, more than 70% of the households had an income of less than the minimal recommendation for appropriate living in Jakarta. Furthermore, the limited household incomes might affect the availability and affordability of nutritious foods and increase child malnutrition resulting in anemia. In this study it was revealed that children aged 6–36 months were at risk of anemia if they had inadequate dietary intake of both macro nutrients (i.e., fats, and protein), and micronutrients (i.e., calcium, iron, zinc, vitamin A, vitamin C, vitamin B6, and vitamin B12), and also if not consuming cow’s milk formula. This showed that anemia among children aged 6–36 months old was not only associated with iron intake. Houghton et al. [17] concluded from a study among children aged 12–23 months in New Delhi that although iron deficiency was found to be the only nutrition factor significantly associated with anemia, most of the children who were classified as anemic were having multiple micronutrient deficiencies, including folate, vitamin D, vitamin B12, zinc, and vitamin A.

There was a negative association between dietary calcium intake and anemia because calcium inhibits iron absorption. However, the results from most multiple-meal studies suggested that calcium would have only a small effect on iron absorption unless habitual calcium consumption was very low. Thus, it was concluded that dietary calcium intake was unlikely to have a biologically significant impact on iron balance, and the mechanism by which calcium reduced iron absorption remained undetermined. It is predicted that the interaction of calcium with other meal components might have a role, for example, with the protein content in dairy milk or with phytate content in bread [18]. Inadequate dietary calcium intake in this study did not exist as one of the determinant factors of anemia.

For vitamin D, Chowdhury et al. [19] reported that vitamin D deficiency was associated with moderate anemia in young children in North India, while the effect was independent of iron deficiency. Up to now, the role of vitamin D in increasing the risk of anemia is still debated and needs to be evaluated in further studies.

Anemia is mostly associated with iron deficiency status. There is a significant positive correlation between dietary iron intake and hemoglobin values among children aged 24–36 months from the same area [20]. This shows the importance of an appropriate dietary intake in the prevention of anemia. However, a study among preschooler children in Brazil found that there was no significant difference between dietary intake of iron, energy, and protein between those children with or without iron depletion or iron deficiency anemia status [21]. This finding suggests that besides paying attention to adequate intake of dietary iron, other factors such as socio-economic of the household and childcare are also important as confounding variables [22].

As mentioned in the Brazilian study [21], inadequate intake of lipids and protein was also associated with anemia. Considering oil intake as an intake of lipid, a community-based cross-sectional study done in South-West Ethiopia found that iron deficiency anemia was significantly lower among subjects consuming oils [22]. Not-consuming protein-source foods, not-consuming dairy products, not-consuming discretionary calories, low family income and intestinal parasitic infections were predictors of IDA [22]. Dietary fat intake contributed as a source of energy and was important for fat-soluble vitamins, namely vitamin A that is known to reduce anemia [23]. With regard to dietary protein intake, it is well-known that protein plays a central role in iron metabolism through transferrin transports and ferritin stores of iron [24].

In this study, inadequate dietary zinc was associated with anemia among children aged 6–36 months old, and it remained as one of the two determinant factors for anemia. The odds of anemia were 3.8 times greater for children with inadequate dietary zinc intake. Dietary zinc intake was also found to be associated with anemia in children less than 24 months of age in a study done in Guatemala, in which the odds of anemia were more than 3 times greater for infants/toddlers with zinc deficiency [25]. Zinc is known as part of more than 100 enzymes and transcription factors involved in growth and cell division, as well as for erythropoiesis, stabilizing erythroid cell membranes, acting as catalyst of the enzyme that modulates erythroid transcriptional gene expression, supports the proliferation of immature erythroblasts, and influences the development of erythroid stem cells. Furthermore, by compromising the function of zinc-dependent antioxidant enzymes, the life span of red blood cells is reduced in the presence of zinc [26].

The odds of anemia in this study were 4.5 times greater for children with insufficient intake of vitamin A. Willows et al., found in their study among preschool children in Yunnan Province, China, that more anemic children had intakes below the estimated average requirement for vitamin A compared to non-anemic children [23]. It is known that besides reducing anemia, vitamin A may also reduce the prevalence of chronic infections, which can result in anemia.

The significant association between inadequate dietary vitamin C intake and anemia could be explained through the fact that vitamin C is known to facilitate ferric ion reduction to ferrous, which is more easily absorbed. Therefore, dietary vitamin C intake is beneficial in preventing anemia [23].

The insufficient dietary intake of vitamins B6 and B12 can be related to anemia because both vitamins are associated with homocysteine concentration. The decrease in those vitamins level correspond with an increase in homocysteine concentrations [26]. Furthermore, increased homocysteine levels are associated with iron deficiency anemia biomarkers, i.e., hemoglobin, hematocrit, and serum ferritin [27].

This study had odds of anemia 8.6 times greater for children not consuming cow’s milk formula. The possible explanation was that cow’s milk formula had been fortified with important micronutrients, including iron, to support child growth and development. Therefore, children that consumed cow’s milk formula had a lower risk of anemia. Cow milk (unmodified) is known to have poor iron content and absorption, low vitamin C content, and in contrast, high casein and calcium content that could negatively influence iron absorption, and thus hemoglobin synthesis [28,29,30].

The present study has several limitations. First, anemia prevalence was only based on measurement of hemoglobin level without assessing other anemia indicator, i.e., MCV, total count of erythrocytes, or reticulocyte count. Second, the semi-quantitative FFQ was adapted from the existing one without validation due to restricted time given by the authority for data collection. Third, the absence of the possibility of checking for under- or over-reporting of nutrient intakes. However, this study had the strength in identifying significant dietary determinant factors of anemia in a homogenous socio-economic condition. This result could be used as a consideration to intervene anemia prevalence in the poor urban area.

## 5. Conclusions

In conclusion, this study shows that almost 30% of infants and toddlers in this selected population suffer from anemia. There are two determinant factors that contribute to the anemic status among children age 6–36 months, i.e., inadequate dietary intake of zinc and not consuming cow’s milk formula. Therefore, the government should continue to assist communities to maintain a diversified dietary intake that will provide an adequate intake of both macro- and micronutrients. Further research must determine the appropriate local diet for children aged 6–36 months, especially for those living in a poor urban area.

## Figures and Tables

**Table 1:** Socio-demographic characteristics of children aged 6–36 months.

Socio-Demographic Characteristics	Total Subject (185)
Age, month	22 (6–36)
Age group, *n* (%)	
6–11 month	27 (14.6)
12–23 month	75 (40.5)
24–36 month	83 (44.9)
Sex, *n* (%):	
Boy	90 (48.6)
Girl	95 (51.4)
Education of Father, *n* (%):	
Up to Junior high school	65 (35.1)
Senior high school and over	120 (64.9)
Education of Mother, *n* (%):	
Up to Junior high school	76 (41.1)
Senior high school and over	109 (58.9)
Occupation of Father, *n* (%)	
Not permanent	114 (61.6)
Permanent	71 (38.4)
Occupation of Mother, *n* (%)	
Not permanent	179 (96.8)
Permanent	6 (3.2)
Household income, *n* (%)	
Less than minimal income	143 (77.3)
Fulfill to minimal income	42 (22.7)

Legend: the population was homogeneous regarding their socio-economic characteristics.

**Table 2:** Feeding practice of children aged 6–36 months (*n* = 185).

Feeding Practice	n (%)
Exclusive BF practice for 6 months, *n* (%)	145 (78.4)
Intake of cow’s formula milk, *n* (%)	117 (63.2)
Taking supplement, *n* (%)	39 (21.1)

**Table 3:** Nutrient intake of children aged 6–36 months (*n* = 185).

Nutrients	Mean ± SD or Median (Min–Max)	Inadequate Intake *n* (%)
Dietary energy intake, in Kcal/day	969.8 (90.5–2230.0)	130 (70.3)
Carbohydrate to total energy, in %	55.3 ± 9.7	147 (79.5)
Fats to total energy intake, in %	32.0 (8.0–51.0)	108 (58.4)
Protein to total energy intake, in %	12.0 (6.0–25.0)	35 (18.9)
Protein intake, in g/kg body weight	2.9 (0.6–8.3)	
Dietary calcium intake, in mg/day	481.5 (35–3381.8)	112 (60.5)
Dietary iron intake, in mg/day	7.4 (0.4–74,0)	89 (48.1)
Dietary zinc intake, in mg/day	4.5 (0.6–54.9)	58 (31.4)
Dietary vitamin A intake, in mcg/day	1021.8 (62.4–7041.4)	37 (20.0)
Dietary vitamin D intake, in mcg/day	2.9 (0–119.8)	172 (93.0)
Dietary B6 intake, in mg/day	0.8 (0.1–119.9)	37 (20.0)
Dietary B9 intake, in mcg/day	132.9 (15.7–597.8)	104 (56.2)
Dietary B12 intake, mcg/day	2.5 (0.2–2004.5)	50 (27.0)
Dietary vitamin C intake, mg/day	60.6 (4.1–445.4)	56 (30.3)

**Table 4:** Hemoglobin and anemia status of children aged 6–36 months (*n* = 180).

Subject’s Sociodemographic Characteristics	*n*	Hemoglobin Level	Anemia Prevalence *n* (%)
Total	180	11.4 ± 1.7	53 (29.4)
Age group			
Age 6–11 month	26	10.9 ± 1.6	11 (42.3)
Age 12–23 month	74	11.5 ± 1.6	19 (25.7)
Age 24–36 month	80	11.3 ± 1.8	23 (28.7)
Sex			
Boy	89	11.3 ± 1.7	26 (29.2)
Girl	91	11.4 ± 1.7	27 (29.7)
Education of Father, *n* (%):			
Up to Junior high school	63	10.9 ± 1.7 *	23 (36.5)
Senior high school and over	117	11.6 ± 1.7	30 (25.6)
Education of Mother, *n* (%):			
Up to Junior high school	74	11.1 ± 1.7	25 (33.8)
Senior high school and over	106	11.5 ±1.7	28 (26.4)
Occupation of Father, *n* (%)			
Not permanent	112	11.3 ± 1.8	33 (29.5)
Permanent	68	11.4 ± 1.6	20 (29.4)
Occupation of Mother, *n* (%)			
Not permanent	174	11.4 ± 1.7	51 (29.3)
Permanent	6	10.7 ± 1.7	2 (33.3)
Household income, *n* (%)			
Less than minimal income	149	11.2 ± 1.7 *	48 (32.2)
Fulfill to minimal income	31	12.0 ± 1.4	5 (16.1)

* *p*-value < 0.05.

**Table 5:** Determinants factors of anemia among the subjects (*n* = 180).

Determinant Factors	*n*	Anemia *n* (%)	*p*-Value	OR (CI95%)	OR (Logistic Regression) *
Energy intake					
Adequate	54	12 (22.2)	0.164		
Inadequate	126	41 (32.5)			
Carbohydrate intake					
Adequate	38	10 (26.3)	0.634		
Inadequate	142	43 (30.3)			
Fats intake					
Adequate	76	14 (18.4)	0.006	2.657	
Inadequate	104	39 (37.5)		(1.316–5.366)	
Protein intake					
Adequate	148	36 (24.3)	0.001	3.526	
Inadequate	32	17 (53.1)		(1.601–7.764)	
Calcium intake					
Adequate	71	9 (12.7)	<0.001	4.663	
Inadequate	109	44 (40.4)		(2.102–10.347)	
Vitamin D intake					
Adequate	13	1 (7.7)	0.074		
Inadequate	167	52 (31.1)			
Iron intake					
Adequate	94	16 (17.0)	<0.001	3.681	
Inadequate	86	37 (43.0)		(1.852–7.315)	
Zinc intake					
Adequate	124	25 (20.2)	<0.001	3.960	4.262
Inadequate	56	28 (50.0)		(2.000–7.842)	(*p* = 0.042)
Vitamin A intake					
Adequate	145	33 (22.8)	<0.001	4.525	
Inadequate	35	20 (57.1)		(2.087–9.811)	
Vitamin C intake					
Adequate	127	29 (22.8)	0.003	2.797	
Inadequate	53	24 (45.3)		(1.415–5.528)	
Vitamin B6 intake					
Adequate	145	36 (24.8)	0.006	2.860	
Inadequate	35	17 (48.6)		(1.334–6.130)	
Folate intake					
Adequate	80	19 (23.8)	0.134		
Inadequate	100	34 (34.0)			
Vitamin B12 intake					
Adequate	133	30 (22.6)	0.001	3.290	
Inadequate	47	23 (48.9)		(1.631–6.637)	
Type of milk consumed					
Cow’s milk formula	113	14 (12.4)	<0.001	9.849	8.651
Non-cow’s milk formula	67	39 (58.2)		(4.695–20.662)	(*p* < 0.001)

Legend: The first OR is the result from the bivariate analyses (chi-square test), while the second OR is the result from the logistic regression analysis, which was performed among those with *p*-value < 0.020 from the bivariate analyses (chi-square test). * Including *p*-value of <0.020 from the bivariate analysis after controlling for sex, father’s education status and household income status.

## Data Availability

No other data are available.
